# Winter and Its Dangers

**Published:** 1879-11

**Authors:** 


*Winter and its Dangers*—By Hamilton Osgood, M. D., Editorial Staff of the *Boston Medical & Surgical Journal.* Publishers: Lindsay & Blackiston, Philadelphia, 1879.

This small but highly interesting and instructive treatise forms one of the Series of American Health Primers; the publication of which is so beneficial to health and comfort.

Commencing with general considerations, in which negligence of health is justly termed sin and stupidity, the author discusses the dangers arising from errors in dress, carelessness and ignorance in bathing, inattention to pulmonary food, danger from over-heated air, indifference to sunshine, sedentary life and neglect of exercise, dangers of school life in winter, winter amusements, etc. The portion of the work relating to proper ventilation of sleeping apartments is of the greatest importance; and this small work is vastly more useful than its size would appear to indicate.

